# An expanded GCaMP reporter toolkit for functional imaging in *Caenorhabditis elegans*

**DOI:** 10.1093/g3journal/jkad183

**Published:** 2023-08-11

**Authors:** Jimmy Ding, Lucinda Peng, Sihoon Moon, Hyun Jee Lee, Dhaval S Patel, Hang Lu

**Affiliations:** Interdisciplinary Bioengineering Graduate Program, Georgia Institute of Technology, Atlanta, GA 30332, USA; Interdisciplinary Bioengineering Graduate Program, Georgia Institute of Technology, Atlanta, GA 30332, USA; School of Chemical & Biomolecular Engineering, Georgia Institute of Technology, Atlanta, GA 30332, USA; School of Chemical & Biomolecular Engineering, Georgia Institute of Technology, Atlanta, GA 30332, USA; School of Chemical & Biomolecular Engineering, Georgia Institute of Technology, Atlanta, GA 30332, USA; Interdisciplinary Bioengineering Graduate Program, Georgia Institute of Technology, Atlanta, GA 30332, USA; School of Chemical & Biomolecular Engineering, Georgia Institute of Technology, Atlanta, GA 30332, USA

**Keywords:** GCaMP, calcium reporters, imaging, mechanosensation, microfluidics, *Caenorhabditis elegans*

## Abstract

In living organisms, changes in calcium flux are integral to many different cellular functions and are especially critical for the activity of neurons and myocytes. Genetically encoded calcium indicators (GECIs) have been popular tools for reporting changes in calcium levels in vivo. In particular, GCaMPs, derived from GFP, are the most widely used GECIs and have become an invaluable toolkit for neurophysiological studies. Recently, new variants of GCaMP, which offer a greater variety of temporal dynamics and improved brightness, have been developed. However, these variants are not readily available to the *Caenorhabditis elegans* research community. This work reports a set of GCaMP6 and jGCaMP7 reporters optimized for *C. elegans* studies. Our toolkit provides reporters with improved dynamic range, varied kinetics, and targeted subcellular localizations. Besides optimized routine uses, this set of reporters is also well suited for studies requiring fast imaging speeds and low magnification or low-cost platforms.

## Introduction

Calcium ions play a significant role in many biological processes, such as apoptosis, transcription, muscle contraction, and neuronal excitability (Clapham 2007). Imaging intracellular calcium flux using genetically encoded calcium indicators (GECIs) is an invaluable tool for in vivo measurement of neural activity. Continual advances in GECIs have resulted in brighter, more sensitive reagents with faster kinetics, such as the GCaMP6 (Chen *et al*. 2013) and jGCaMP7 (Dana *et al*. 2019) families of fluorophores. These higher signal-to-noise fluorophores and advances in optical microscopy have allowed more reliable measurement of neural activity during demanding experiments, such as recording from many neurons in parallel (Ahrens *et al*. 2013; Kato *et al*. 2015; Li *et al*. 2017; Wirak *et al*. 2022), neural processes (Hendricks *et al*. 2012; Parker *et al*. 2016; Liu *et al*. 2018), and whole-brain imaging in freely behaving animals (Nguyen *et al*. 2016; Venkatachalam *et al*. 2016; Kim *et al*. 2017; Zong *et al*. 2022).

In the model organism *Caenorhabditis elegans* (*C. elegans*), GCaMP has been widely used to determine the roles of individual neurons within the nervous system. For example, the expression of cytosolic GCaMP in different classes of neurons has mapped their activities to specific stimuli (Zaslaver *et al*. 2015), mapped circuits that drive olfactory behaviors (Chalasani *et al*. 2007; Ha *et al*. 2010), shown experience-dependent changes in neural activity (Ha *et al*. 2010; Hong *et al*. 2017; Wu *et al*. 2023), and verified the roles of key hub neurons in sustaining behavioral states (Flavell *et al*. 2013) among many applications. Additionally, it has been shown that subcellular (Hendricks *et al*. 2012) and multicellular (Gordus *et al*. 2015; Kato *et al*. 2015) calcium activity play important roles in the neurophysiology of *C. elegans*. Such studies impose greater performance demands on optical microscopy systems as they confine fluorophores to specific cell regions, such as nuclei for multicell imaging and processes for subcellular imaging, which come with reduced signal (or signal-to-noise ratio). The newer generation GCaMP reagents (GCaMP6 and jGCaMP7) (Chen *et al*. 2013; Dana *et al*. 2019) can alleviate some of these technical challenges. However, *C. elegans*-optimized versions of GCaMP6f, jGCaMP7s, and 7f remain publicly unavailable, and the in vivo properties of jGCaMP7 variants have not been fully characterized in *C. elegans*.

To address the *C. elegans* community's need, we have made a toolkit of codon-optimized reagents that includes four variants with distinct kinetic profiles (GCaMP6f, 6s, jGCaMP7f, 7s), each available with three subcellular localizations—cytosolic, nuclear, and membrane-targeted. The kinetic variants offer flexibility in balancing brightness and speed for different experiments. At the same time, the subcellular localization tags enable imaging of calcium flux in specific parts of a neuron. For example, membrane localization allows neurite recording, while nuclear localization enables the separation of signals from multiple neurons recorded in parallel. Additionally, to facilitate the detection of neurons and neurites during fluctuations in GCaMP brightness and reduce motion artifacts when processing data, our reporters are bicistronic and express the bright red fluorophore mScarlet-I (Bindels *et al*. 2017) in tandem with each GCaMP variant.

To compare and contrast the properties of each GCaMP variant in vivo, we generated single-copy knock-ins driven by the same promoter at the same genomic locus. We then recorded the mechanosensory responses from the gentle touch neuron ALM in strains carrying each variant from our toolkit. We also demonstrate the efficacy of these reagents in recording mechanosensory responses from the soma at low magnification and neural processes at high magnification. Finally, by making our library of reagents publicly available, we hope to facilitate the investigation of subcellular and multicellular calcium dynamics amongst the *C. elegans* research community.

## Materials and methods

### Generation of GCaMP variants

We took an in silico approach to optimizing the coding sequence (CDS) of GCaMP6s (Chen *et al*. 2013) for efficient expression in *C. elegans*. First, we created a nuclear-localized version of the fluorophore by adding the SV40 nuclear localization sequence (NLS) to its N-terminus and an *egl-13* NLS at the C-terminus (Lyssenko *et al*. 2007). Next, we optimized this modified sequence for the worm genome using the *C. elegans* codon adapter (Redemann *et al*. 2011), which also inserted artificial introns into the CDS to enhance expression in vivo. The optimized sequence was then synthesized as a gene fragment (Integrated DNA Technologies) along with a fragment containing the *gpd-2/gpd-3* operonic linker (Huang *et al*. 2001) with overlaps with the last exon of our nuclear-localized GCaMP6s and the first exon of our existing nuclear-localized mScarlet-I gene (Stevenson *et al*. 2023). These two gene fragments were used directly for DNA assembly (New England Biolabs) into pDSP9, our mScarlet-I plasmid, to obtain pDSP23, a promoterless version of the bicistronic construct containing nuclear-localized GCaMP6s linked to nuclear-localized mScarlet-I (Supplementary Fig. 1).

The promoterless vectors for nuclear-localized GCaMP6f, 7s, and 7f variants were derived by site-directed mutagenesis (NEB Q5 Site-Directed Mutagenesis Kit) of the GCaMP6s sequence in pDSP23 to reproduce the mutations described for GCaMP6f (pDSP31) (Chen *et al*. 2013) and jGCaMP7s (pDSP32), 7f (pDSP33) (Dana *et al*. 2019). The cytosolic GCaMP6s, 6f, jGCaMP7s, and 7f variants (pHJL1-4, respectively) were derived from the nuclear-localized versions via PCR and DNA assembly to remove the SV40 and *egl-13* NLS (Supplementary Fig. 1). The membrane-localized GCaMP6s, 6f, jGCaMP7s, and 7f variants (pSiM1-4, respectively) were also derived from the nuclear-localized versions using the same methodology to remove the SV40 and *egl-13* NLS and add the C-terminal *ras-2* CAAX domain (Chen *et al*. 2013; Hisamoto *et al*. 2016) to both the GCaMP and mScarlet-I genes (Supplementary Fig. 1). All vectors were sequenced to verify correct assembly before further use. The primer sequences used for all constructions are provided in Supplementary File 1.

### Single-copy transgenesis of *C. elegans*

We created single-copy knock-ins of each of our constructs driven by the *mec-7* promoter, expressed in the gentle touch neurons (Hamelin *et al*. 1992), using mos-mediated transgenesis (Frokjaer-Jensen *et al*. 2012). The *mec-7* promoter was amplified from genomic DNA. This amplicon was assembled along with the bicistronic GCaMP-mScarlet-I construct of each variant into pDSP2, a kanamycin-resistant version of the pCFJ350 vector (Frokjaer-Jensen *et al*. 2012). Oligo sequences for all PCRs are given in Supplementary File 1. Transgenic single-copy integrants were obtained following the MosSCI protocol (Frokjaer-Jensen *et al*. 2012). Briefly, QL74, a gift from QueeLim Ch’ng, was used for all construct injections. Single-copy integrants were screened using *peel-1* negative selection and loss of extrachromosomal co-injection markers. The resulting strains are listed in Supplementary Table 1.

### Imaging/data collection

All functional imaging experiments were performed using standard wide-field fluorescence microscopy on the system described in Cho *et al*. (2017). Briefly, 2-day-old adult worms are loaded into and acclimated to the microfluidic device and imaging light for 2 min. Baseline neural fluorescence was recorded for 10 s before delivering a 3-s 2.5 Hz mechanical stimulus. The neural activity following the mechanical stimulus was recorded for 60–90 s. All functional imaging was performed on a Leica DMIRB inverted microscope with a 40 × air objective (N.A. 0.75) unless otherwise specified. Low magnification dynamics (Fig. 3) were recorded using a 10 × (N.A. 0.22) air objective. Video recordings were performed on a Hamamatsu EM-CCD with 100 ms exposure. Simultaneous two-color imaging was acquired with a DV2 beam splitter (Photometrics) and GFP/RFP filter set (520 and 630 nm) with the excitation coming from a filtered projector source (475 and 568 nm) (Stirman *et al*. 2011).

### Analysis

**Fig. 1.**
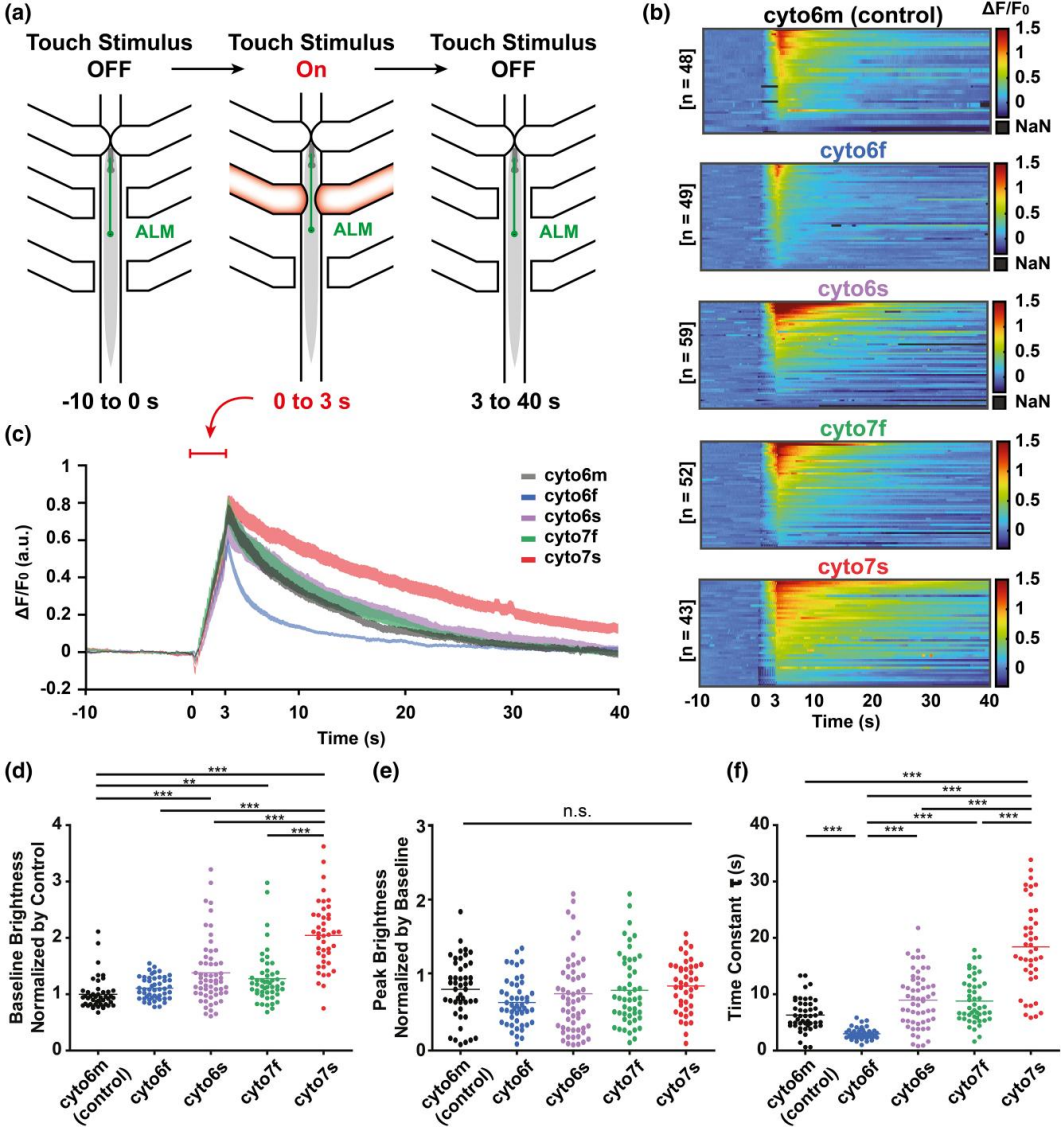
jGCaMP7 performs better than GCaMP6 in vivo. a) Visual representation of the touch stimulation procedure. Touch stimuli are simulated with 3-s 2.5 Hz mechanical presses at the 0-s mark. b) Individual fluorescence intensity over time, with stimulus delivered at *t* = 0 s, are shown for each developed cytosolic localized GCaMP strain and the existing AQ3236 strain, which is used for comparison. c) GCaMP brightness, normalized by prestimulus baseline, increases then decreases in the mechanosensory neuron over time, where a controlled mechanical stimulus is applied from 0 to 3 s. The amplitude of brightness relative to baseline and the time to relax back to baseline varies depending on the GCaMP variant. d) Baseline brightness of GCaMP of each developed cytosolic variant is compared to a previously developed GCaMP6m cytosolic reporter strain, normalizing all values by the average baseline brightness of the comparison strain. Notably, the newly developed 7f and 7s GCaMP variants are significantly brighter than the GCaMP6m strain. e) Peak brightness of GCaMP for each of the GCaMP variants is compared. f) The time constants of the decays of each GCaMP variant are compared. Notably, 7s is found to decay significantly slower than all other strains, and 6f is significantly faster than all other variants. The Kruskal–Wallis test was performed, followed by Dunn's multiple comparison test (**P < 0.01, ***P < 0.001).

**Fig. 2.**
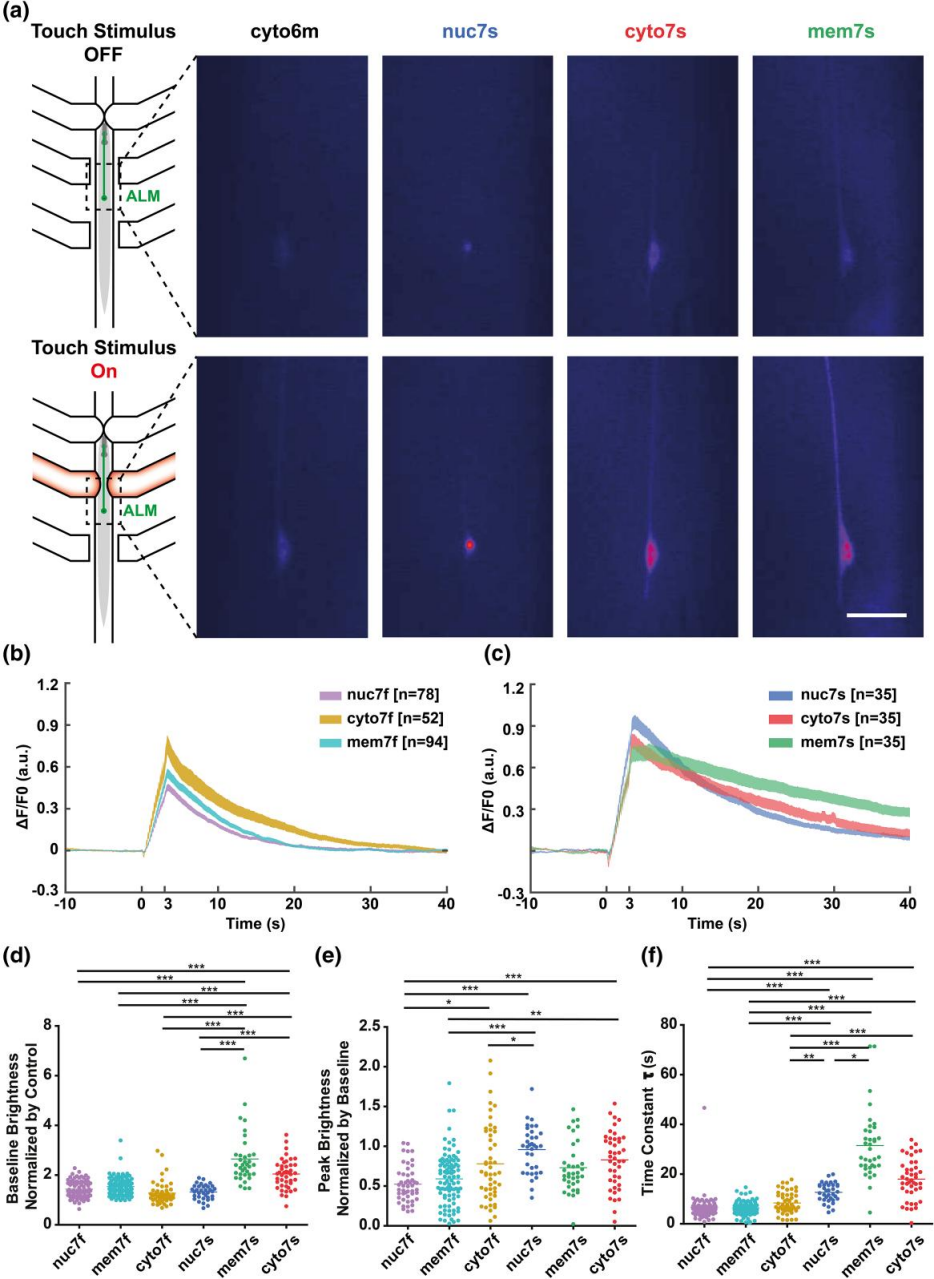
Quantitative comparison between different subcellular localizations. a) Mechanical stimulus is delivered via pneumatic valves, leading to an increase in neuronal fluorescence. Example baseline (top) and excited (bottom) fluorescent images are shown for GCaMP6m comparison strain and the cytosolic, nuclear, and membrane subcellular localizations for jGCaMP7s. Scale bar: 25 μm. b, c) Average change in fluorescence over time normalized by baseline activity, with stimulus occurring at *t* = 0 s, for all three localizations of jGCaMP7f (b) and 7s (c). The measured brightness increases after stimulus delivery before reaching a peak and returning to baseline. d) Comparison of baseline brightness normalized by control strain (AQ3236, GCaMP6m) for all subcellular localizations of jGCaMP7f and jGCaMP7s. All strains are found to be, on average, brighter than the control strain. The nuclear-localized strains are found to be dimmest, while membrane-bound jGCaMP7s is found to be the brightest. e) Peak GCaMP brightness normalized by baseline brightness for each subcellular localization of jGCaMP7f and 7s. Generally, the jGCaMP7f variants are seen to have a somewhat lower dynamic range. f) Comparison of the speed of decay for each subcellular localization. All localizations of the jGCaMP7f variants are significantly faster than all 7s variants, with membrane 7s decaying at the slowest rate and membrane 7f with the most consistently fast decay. The Kruskal–Wallis test was performed, followed by Dunn's multiple comparison test (*P < 0.05, **P < 0.01, ***P < 0.001).

**Fig. 3.**
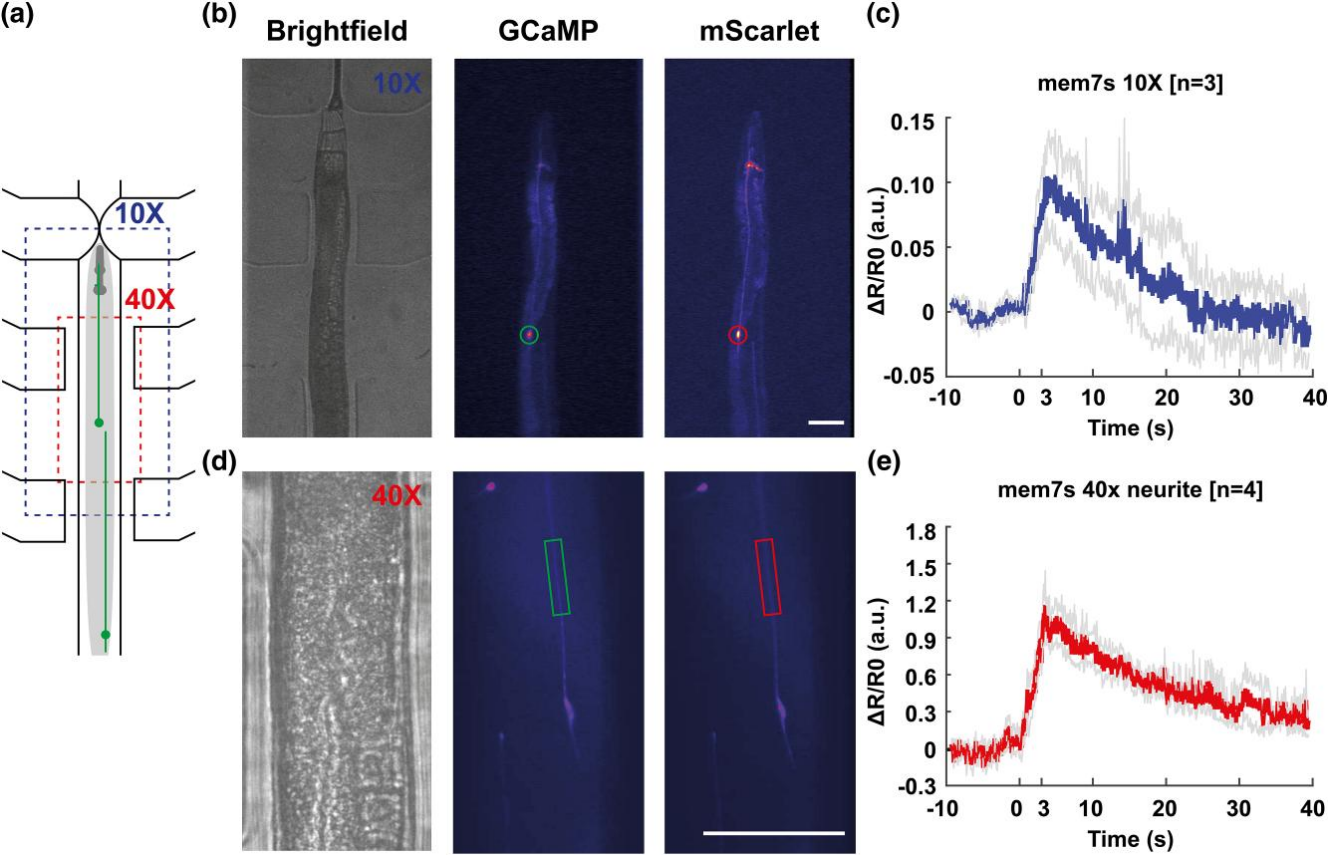
Low magnification and neurite functional imaging are demonstrated using newly developed strain. a) Diagram demonstrating increasing field of view at lower 10 × magnification compared to the 40 × magnification used in previous figures. b) Brightfield (left), fluorescent GCaMP (middle), and mScarlet (right) images taken at lower magnification, with nerve ring and ALM neuron body visible. Circles indicate ROI for the calculation of fluorescent intensity. Scale bar: 50 µm. c) Functional imaging is still possible at 10 × magnification using our microscopy platform and the newly developed membrane-localized GCaMP7s reporter strain, with a significant increase in normalized fluorescence compared to baseline activity. d) Brightfield (left), fluorescent GCaMP (middle), and mScarlet (right) images taken at standard magnification demonstrate that the neuron process is clearly visible. Boxes indicate ROI for the calculation of fluorescent intensity. Scale bar: 50 µm e) Functional imaging of neurite is demonstrated to be possible for the membrane jGCaMP7s strain under these imaging conditions.

Fluorescence intensities for each frame were obtained as previously described (Cho *et al*. 2017). Briefly, the GCaMP-only (Δ*F*/*F*_0_) and ratiometric (GCaMP divided by TagRFP or mScarlet, Δ*R*/*R*_0_) fluorescence intensity values were computed using a customized MATLAB script. While Δ*R*/*R*_0_ is desirable to correct for potential motion artifacts in most imaging experiments (such as those shown in Fig. 3, where Δ*R*/*R*_0_ is used), we report Δ*F*/*F*_0_ in Figs. 1 and 2 to allow the most direct comparison of GCaMP performance across strains expressing different red fluorophores. The existing strain (AQ3236) expresses noncodon-optimized TagRFP (dimmer), while the strains expressing the newly developed reporters express codon-optimized mScarlet-I (brighter). While Δ*R*/*R*_0_ could be skewed by these differences in red brightness, preventing direct GCaMP comparison, Δ*F*/*F*_0_ depends on GCaMP brightness alone. The baseline fluorescence values (*R*_0_, *F*_0_) were calculated using the frames prior to the stimulus.

### Behavior assay

To assess the behavioral responses of the new GCaMP strains, N2 and GT350 (membrane-bound GCaMP7s) adult worms were transferred to an unseeded plate. After at least 15 min acclimation, worms were stimulated with an eyebrow hair to the anterior half. The number of reversals was recorded.

## Results and discussion

### Design of the transgenes

To provide a toolkit of GCaMPs tailored to *C. elegans* neuroscience research, we modified the most frequently used variants of GCaMP from mammalian studies. The *C. elegans*-specific toolkit features four variants with different response kinetics, each available with three different subcellular localizations. In addition to cytoplasmic fluorophores, we have created versions with localization to the nucleus and cell membrane to accommodate expansive imaging conditions. For example, the nuclear-localized expression may be helpful in imaging dense cell populations, such as in whole-brain imaging. The membrane-localized fluorophores can enable visualization of the calcium dynamics in the neurites and increase the overall brightness of the neuron for low-magnification imaging by encouraging greater labeling of the neuronal soma. Further, we have incorporated two kinetic variants of GCaMP for different imaging needs: an f (fast) variant for faster rise and decay kinetics and an s (slow) variant for higher sensitivity While the s variants typically offer higher signal-to-noise ratios, the f variants are useful in applications where responses to rapidly changing stimuli or interneuronal signaling must be observed.

To achieve the maximum possible signal-to-noise ratio, we first optimized the codon usage of GCaMP6s for the *C. elegans* genome. In addition, we also added three artificial introns into the CDS, as these are known to enhance transgene expression (Okkema *et al*. 1993). These modifications increase the number of *C. elegans* optimal codons from ∼27% in the original sequence to 85% while avoiding repetitive DNA sequences that could promote transgene silencing. After creating our worm-optimized GCaMP6s, we used it as a template for site-directed mutagenesis to generate GCaMP6f (Chen *et al*. 2013) and jGCaMP7s and 7f, once their sequences were publicly available (Dana *et al*. 2019). Many neural activity imaging experiments also use a red fluorophore, whose brightness is not linked to calcium levels, as a reference within each cell to help correct motion artifacts and facilitate the detection of neurons and processes with dim baseline GCaMP fluorescence during imaging and data processing. To employ this strategy in our toolkit, we made our constructs bicistronic by including our codon-optimized mScarlet-I gene (Stevenson *et al*. 2023) linked to the 3′ end of GCaMP gene using the *gpd-2/gpd-3* operonic linker sequence that promotes trans-splicing (Huang *et al*. 2001). The bicistronic expression of GCaMP and mScarlet-I ensures similar protein levels within each cell, allowing the calcium-insensitive red fluorophore to be used for ratiometric normalization of the dynamic GCaMP signal. While we expect that co-expression of mScarlet-I will be desirable for most *C. elegans* imaging applications to correct for motion artifact and enable robust detection of neurons and processes with dim GCaMP fluorescence, the mScarlet-I could also easily be removed when cloning a promoter into our reporters by designing an additional set of primers that directly assembles the 3′ end of GCaMP to the 5′ end of the *tbb-2* 3′ UTR and running a three-fragment assembly (promoter, GCaMP, *tbb-2* 3′ UTR + backbone), if GCaMP expression alone is needed.

### jGCaMP7 performs better than GCaMP6 in vivo

To compare and contrast the properties of *C. elegans*-optimized GCaMP6 and jGCaMP7 and to highlight the differences and utility of in vivo characteristics of the slow and fast variants, we expressed these single-copy transgenes in the mechanosensory neurons ALM, AVM, PLM, and PVM using the *mec-7* promotor (Hamelin *et al*. 1992. We compare the s (slow) and f (fast) variants of GCaMP6 and jGCaMP7 because they have the maximum brightness and fastest decay dynamics, respectively. The ALM and AVM neurons are the sensory neurons responsible for responding to gentle touch in the anterior half of the body. To compare the functional activity of several GCaMP variants (6s, 7s, 6f, 7f), we used previously developed microfluidic actuator methods (Cho *et al*. 2017) to test the in vivo function of the reporters in ALM in response to consistent mechanical stimuli. The microfluidic system has an on-chip pneumatic actuator that applies pressure to locations along the worm body to simulate touches on demand (Fig. 1a). In addition, we compared these GCaMP variants against an existing state-of-the-art GCaMP6m reporter strain, AQ3236: *ljSi2 [mec-7::GCaMP6m::SL2::TagRFP + unc-119(+)] II; unc-119(ed3) III* (Cho *et al*. 2017). For this comparison, we use a standardized assay in which the stimulus is delivered from *t* = 0 to 3 s. The magnitude of the stimulus is calibrated before each trial to maintain consistency between trials and devices. The imaging conditions, including camera setting and excitation power, were also standardized according to previously established methods (Cho *et al*. 2017). To compare the different localizations and GCaMP variants, we chose imaging conditions that allow for the visualization of fluorophores without pixel saturation while recording the brightest variants. The imaging conditions can be further optimized depending on which variant the user ultimately chooses. Under these stimulus and imaging conditions, we find that the GCaMP variants behave as expected in vivo as previously reported in vitro (Dana *et al*. 2019).

The results of the mechanical stimulation assay demonstrate that the new GCaMP reporter strains cover a range of properties, such as peak brightness and decay time, while performing as well as or better than existing state-of-the-art reporters. Figure 1b shows heatmaps of the fluorescence change for many individuals carrying each reporter responding to the same mechanical stimuli. While all populations are shown to have some individuals with a large increase in fluorescence, the jGCaMP7s and 7f populations have much more activity on a per-individual basis compared to their GCaMP6 counterparts. Figure 1c shows the average fluorescence for each population, normalized by their baseline brightness over time. The fluorescence increases from 0 to 3 s when the stimulus is applied and then returns to the baseline brightness. The normalized peak brightness, representing dynamic range, is similar between all strains. However, when comparing the rate of decay, GCaMP6f is notably faster, and jGCaMP7s is slower than the other strains (Fig. 1c). It is important to note that the amplitude of the peaks, while a fair comparison between strains, is not a representation of the brightest output these fluorophores can achieve in response to other stimuli. The mechanical stimulus of the ALM, which is known to also respond to light stimulation, has been reported to produce around one-fifth of the amplitude of a light response (Nekimken *et al*. 2017). Furthermore, as we have used MosSCI to obtain single-copy integrants, the copy number can be increased further to increase signal and to tune brightness to suit a particular use case.

Comparing baseline brightness and decay rate of the fluorophores shown in Table 1 points to their potential for different experimental uses. When comparing baseline brightness, the jGCaMP7 variants perform significantly better than AQ3236 and the codon-optimized GCaMP6 strains (Fig. 1d, Supplementary Fig. 2). All strains except the optimized GCaMP6f were significantly different in baseline brightness compared to AQ3236, and jGCaMP7s is significantly brighter than all other fluorophores. When comparing the peak brightness of each strain normalized by their baseline brightness, there is no significant difference between the fluorophores (Fig. 1e). This means that, without normalization, the fluorophores with a higher baseline brightness also have a higher peak brightness. As for rates of decay, the jGCaMP7s strain is shown to have the largest time constant, i.e. it is slowest to return to baseline brightness (Fig. 1f), while GCaMP6f is significantly faster than the other variants. For example, the optimized jGCaMP7s has the highest baseline brightness (105% increase from unoptimized 6 m) but also the slowest kinetics (*t*_1/2, fall_ = 18 ± 8 s). The optimized GCaMP6f has the lowest baseline brightness (11% increase from unoptimized 6 m) but the fastest kinetics (*t*_1/2, fall_ = 2.7 ± 2.0 s).

**Table 1.** Brightness and decay speed of cytosolic GCaMP variants.

GCaMP variant	Baseline brightness normalized to 6m	τ (s)
6m	1 ± 0.28	6.3 ± 2.9
6s	1.45 ± 0.57	8.4 ± 5.0
7s	2.05 ± 0.60	18.0 ± 8.1
6f	1.11 ± 0.20	2.7 ± 2.0
7f	1.28 ± 0.46	8.5 ± 4.1

The high baseline brightness of jGCaMP7s combined with its slow decay time makes it best suited for situations where it is not required for the fluorophore to decay quickly or if a slow decay rate is desirable. It is also useful when the highest possible fluorescence intensity is required, such as imaging at low magnification or lower excitation power. On the other hand, jGCaMP7f is shown to be as fast as the control strain and brighter, allowing experiments to be performed at higher camera speeds. The data indicate that jGCaMP7f will be useful when both high temporal resolution and signal are required. Its peak brightness is significantly greater than that of jGCaMP6f, and its decay rate is about 2 times faster than jGCaMP7s (Fig. 1d–f). These properties are useful for measuring neuronal activity at faster time scales, such as responses to a fast, time-varying stimulus. GCaMP6f has the fastest decay rate but has a tradeoff of low dynamic range, which may prevent its use in measurements of low-magnitude stimuli.

### Subcellular localization provides a toolkit for multiple applications

To compare the GCaMP localization, we used the same neurons and measured the baseline and excited fluorescence (Fig. 2a). GCaMP localized to the nucleus could be best used for multicell imaging of a cluster of neurons that would be otherwise unresolvable, e.g. as in whole-brain imaging (Kato *et al*. 2015; Nguyen *et al*. 2016; Venkatachalam *et al*. 2016). Membrane-bound GCaMP may allow for a better study of compartmentalized or subcellular calcium signaling, e.g. in neurites. For example, it could be used to examine local sensory input, e.g. during proprioception (Tao *et al*. 2019).

Next, we characterized and compared different subcellular localizations by imaging their functional response using the standardized assay described above (Fig. 2b–f). We again used an existing state-of-the-art reporter strain, AQ3236 (Cho *et al*. 2017) as a control for comparison with the optimized variants. By examining baseline brightness normalized by the average brightness of the control strain (Fig. 2d), we observed that all new jGCaMP7 variants are brighter than the previously used control strain. The membrane jGCaMP7s is the brightest, with some extreme outliers over 6 times brighter than the control. The rest of the strains are closer in brightness, with general trends of jGCaMP7s being brighter than jGCaMP7f regardless of localization and the membrane and cytosolic localization being brighter than the nuclear-localized strain. Dynamic range is demonstrated by measuring neuronal response to a stimulus delivery normalized by the baseline brightness (Fig. 2e). Again, the jGCaMP7s strains are shown to have a higher dynamic range than their jGCaMP7f counterparts for each localization. Nuclear-localized jGCaMP7f demonstrated the worst range at around a 50% increase from baseline brightness, whereas the nuclear and cytosolic localized jGCaMP7s variants demonstrated a ∼100% increase from baseline. The membrane-bound strains demonstrate a slightly lower dynamic range comparatively. The decay dynamics were also characterized (Fig. 2f), and, as expected, the jGCaMP7s variants are slower to decay compared to their jGCaMP7f counterparts. Notably, membrane jGCaMP7s has the longest decay from the excited state, whereas all three jGCaMP7f localizations decay more rapidly. We speculate that the longer decay time of membrane-localized jGCaMP7s might be due to intracellular gradients in calcium concentration during the transport of calcium out of the cell. The sensitivity of the membrane-localized jGCaMP7s might potentially be capturing these gradients as calcium exits through the membrane, extending the GCaMP signal compared to other variants and localizations. This extended signal could be useful in applications with low framerate, such as volumetric imaging. However, there may be scenarios where faster dynamics and more sensitivity are demanded, and the GCaMP6f or jGCaMP7f variants would be more beneficial. All strains are plotted together and statistically compared in Supplementary Fig. 3, Supplementary File 2, and Supplementary Table 2.

Results presented thus far demonstrate that although nuclear localization decreases the imaging area for neurons and can be beneficial when imaging highly clustered neurons, it also decreases baseline brightness. In contrast, membrane localization increases the baseline brightness by enhancing neuronal soma and process labeling (Supplementary Fig. 2). This increase in fluorophore labeling the soma is beneficial for obtaining higher signal when imaging at lower magnification; further, membrane localization would allow small structures such as neurites to be imaged. These properties make this localization scheme best suited for systems requiring neuron tracking since the higher baseline brightness reduces the chances of the ROI being lost or confused with autofluorescence if a somewhat lower signal-to-noise ratio can be tolerated.

To ensure that the fluorophores do not induce adverse effects, we tested the behavioral response of the brightest strain, expressing membrane jGCaMP7s. Gentle touch has been traditionally assayed based on reversals in response to stroking with an eyebrow hair (Chalfie and Sulston 1981; Chalfie *et al*. 2014). We recorded the reversal rate for N2 and membrane jGCaMP7s strain, and noted that both had a reversal rate of 97 ± 1% (3 trials, *n* > 50 per trial for both strains). The data clearly demonstrate that even the brightest fluorophore is unlikely to induce adverse effects on the animal's ability to sense stimuli.

### New GCaMP variants allow for novel opportunities in functional imaging

Functional imaging at low magnification opens up new opportunities to, for example, record more accurate neuronal activity during behavioral tracking or image the neural activity of many animals simultaneously using a single objective during a population-wide stimulus, such as a chemical cue. Our newly developed reporter strains allow for lower magnification imaging because of their increased brightness. We demonstrate this by recording the fluorescence of the ALM neuron tagged with membrane-localized jGCaMP7s while it is undergoing the same stimulus regime described above, using a lens with much lower magnification and NA (10×, N.A. 0.22) compared to the objective used in the previously described assay (40×, N.A. 0.75) (Fig. 3a). The baseline fluorescence of the gentle touch neurons is bright enough to easily identify through the eyepiece by eye (Fig. 3b). This brightness facilitates proper identification and adjustment of the optical focus of specific neurons at low magnification. We further demonstrate functional imaging in a mechanically stimulated neuron is possible at this magnification, which has not been shown before. The brighter membrane 7s fluorophore allows observation of a significant increase in fluorescence (Fig. 3c) detectable even at low magnification, with a low NA (0.22) air objective and using relatively low excitation from an LCD projector source (Stirman *et al*. 2011) as opposed to a laser. In comparison, 7f or AQ3236 are too dim to image under the same condition.

The subcellular localization of GCaMP also allows for the investigation of intracellular processing or local cellular response. Membrane-bound GCaMP allows us to examine how information is passed from the neurite to the cell body. Our membrane-bound GCaMP strain has a high enough signal-to-noise ratio in the neurite of the mechanosensory neurons to quantify calcium transients in the neurite. We demonstrate this by analyzing the neurite's response to mechanical stimulus in a region of the neurite that is 60–100 μm (150–250 pixels) anterior to the cell body in a few example videos (Fig. 3d). Neurite expression is consistently good in the membrane-bound GCaMP strain; however, further experiment optimization is needed to track and keep the much smaller neurite in focus. While this portion of the process is not visible in the cytosolic and nuclear localizations (Supplementary Fig. 4), we can extract robust GCaMP responses in the membrane-bound localization based on the segmentation of the red channel. Using the same procedure as previously described, we see that the half-life of the signal in the neurite is 16.4 s (Fig. 3e), approximately half of the 31 s half-life observed for the cell body, shown in Fig. 2f. We speculate that the difference in decay time between neurite and cell body may be due to a variety of reasons: the cell body may be integrating the mechanosensory signal from the neurite, the neurite and cell body differ in surface-to-volume ratio and have different geometries, and they possess distinct membrane organelles that regulate Ca2+ dynamics.

This work compares GCaMP6s, 6f, and jGCaMP7s, 7f variants with three different subcellular localization (nuclear, cytosolic, and membrane) in *C. elegans*. We showed both jGCaMP7 variants are brighter than the GCaMP6 counterparts. In vivo results for mammalian data showed that the decay time of GCaMP6f is faster or similar to jGCaMP7f when there is a large signal (greater than 40 action potentials) (Dana *et al*. 2019). In our *C. elegans* data, GCaMP6f shows faster decay rates than jGCaMP7f. However, jGCaMP7f has higher peak brightness (*F*/*F*_0_) than GCaMP6f, so there seems to be a tradeoff between signal and decay rate in *C. elegans*. A similar tradeoff exists among the slow variants (6s and 7s), with jGCaMP7s being slower but brighter and GCaMP6s being faster with less signal. Across generations, GCaMP6s and jGCaMP7f appear to have similar kinetics and brightness when expressed in vivo. We also found that membrane localization provides a greater signal in *C. elegans* due to fluorophore labeling a greater volume of the neuron (both in the soma and processes). Nuclear localization of the protein allows for neurons in a dense population to be distinguished, but our results indicate that this decreases the signal. While the Δ*F*/*F*_0_ was not significantly different between localizations, the effect of localization on the volume of the neuron labeled by a fluorophore and raw fluorescence values means that the overall signal is higher (larger area and larger photon count) in membrane-localized variants. This enhancement facilitates easy identification of the neuron and enables calcium recording even in low magnification and low NA conditions. Furthermore, the ability to image neural activity in freely moving worms has often required custom-built microscopes to get the magnification necessary to record the low signal. In sparsely labeled neurons, it would be advantageous to use membrane-bound GCaMP, as the greater signal allows for lower magnification to be used.

In addition to providing a readily usable set of reporters for the *C. elegans* research community, our toolkit provides a platform for further optimization to meet evolving community needs. For example, as new GCaMP variants continue to be developed and improved, our toolkit can increase the accessibility of making *C. elegans* codon-optimized reporters using site-directed mutagenesis. Rather than fully synthesizing the optimized sequence, the mutations described for a new variant can be applied to the appropriate reporter from our toolkit. For example, the GENIE group at the HHMI Janelia research campus has recently developed its next-generation jGCaMP8 series of reagents (Zhang *et al*. 2023). Our toolkit could be used to obtain *C. elegans* codon-optimized jGCaMP8 variants—as the currently available reporters are optimized for *Drosophila* and mammals—through site-directed mutagenesis of our GCaMP6s plasmids in our toolkit, which could result in reporters with even faster kinetics and higher calcium sensitivity than jGCaMP7f. It is interesting to note that the baseline brightness of the jGCaMP8 variants also appears to be species-dependent, with jGCaMP8 having high baseline brightness in mammals (relative to jGCaMP7f) but lower baseline brightness in *Drosophila*, requiring co-expression of a red marker for some imaging studies (Zhang *et al*. 2023). This work demonstrates the use of codon optimization to improve expression and brightness in *C. elegans*. Other model organisms utilizing GCaMP may also benefit from codon optimizing their GCaMP of choice, as it will likely improve performance and brightness.

## Supplementary Material

jkad183_Supplementary_Data

## Data Availability

Strains and plasmids are available upon request. The authors affirm that all data necessary for confirming the conclusions of the article are present within the article, figures, and tables. GCaMP traces were analyzed using custom code (https://github.com/lu-lab/GCaMP_video_analysis). Supplemental material is available at G3 online.
