# Supplementary material for: An expanded GCaMP reporter toolkit for functional imaging in Caenorhabditis elegans
Source: G3 (Bethesda). 2023 Aug 11;13(10):jkad183. doi: 10.1093/g3journal/jkad183 (PMC10542313; doi:10.1093/g3journal/jkad183)
Supplement: jkad183_Supplementary_Data [file jkad183_supplementary_data.zip › Figure_S1_G3-2023-404350.pdf]

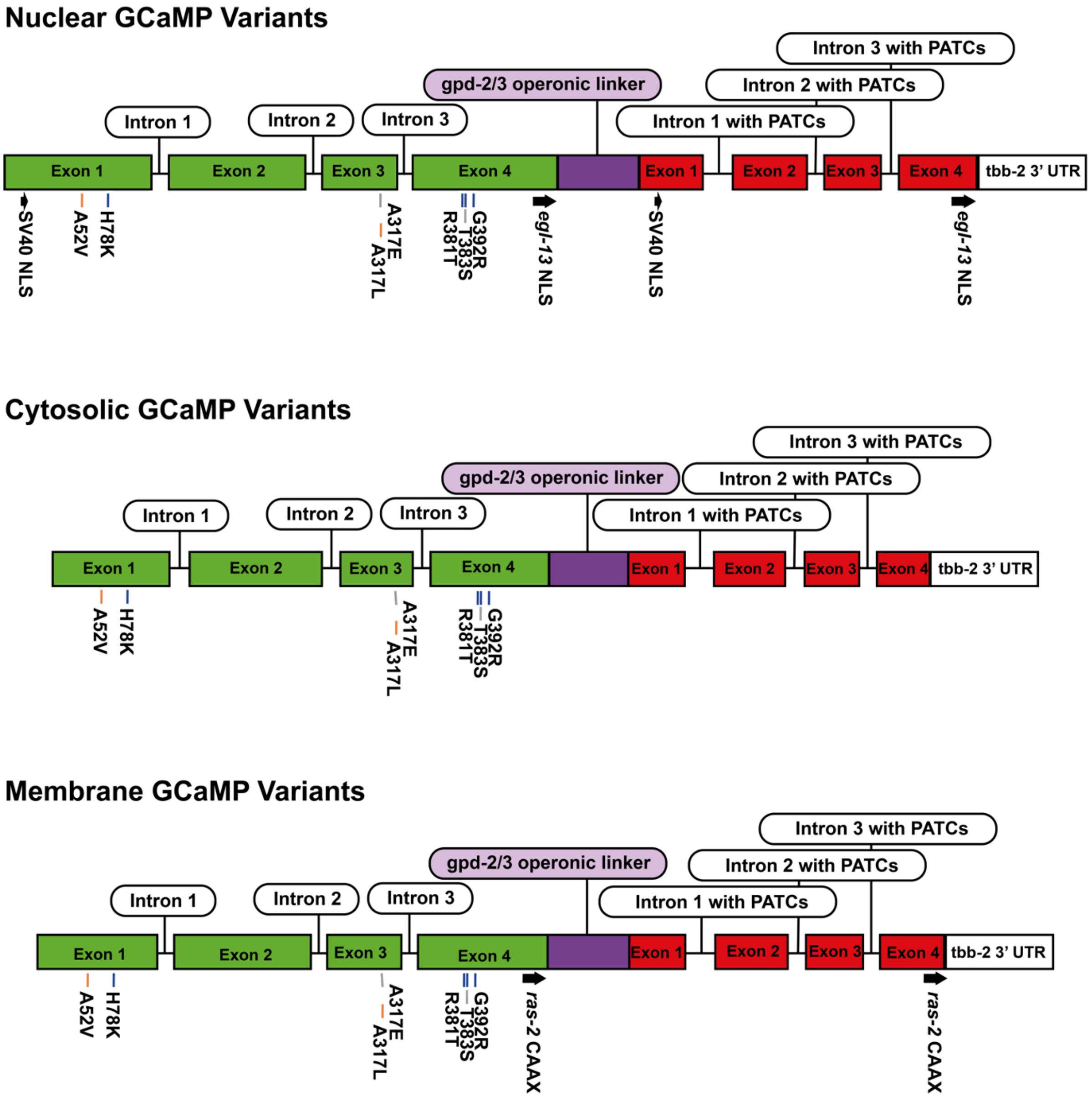


**Figure S1: *C. elegans*-optimized GCaMP reporter toolkit.** Schematic representation of the codon-optimized bicistronic GCaMP and mScarlet-I reporter genes. The exons for the GCaMP and mScarlet-I are color-coded in green and red, respectively. Amino acid substitutions resulting in the 6f (grey), 7s (orange), and 7f (blue) variants from GCaMP6s are indicated below the schematic for each variant. For convenience, we use the numbering scheme used in Dana et al, (2019). (Top) Nuclear-localized variants utilize two NLS sequences in each fluorophore, with the SV40 and *egl-13* NLS encoded at the 5' and 3' ends of the coding sequence, respectively. (Middle) Cytosolic variants were created by removing the NLS sequences from their nuclear-localized counterparts (see Methods). (Bottom) Membrane-localized variants were created from their cytosolic counterparts through the addition of a CAAX domain from the *ras-2* gene (see Methods).
