# Supplementary material for: An expanded GCaMP reporter toolkit for functional imaging in Caenorhabditis elegans
Source: G3 (Bethesda). 2023 Aug 11;13(10):jkad183. doi: 10.1093/g3journal/jkad183 (PMC10542313; doi:10.1093/g3journal/jkad183)
Supplement: jkad183_Supplementary_Data [file jkad183_supplementary_data.zip › Figure_S2_G3-2023-404350.pdf]

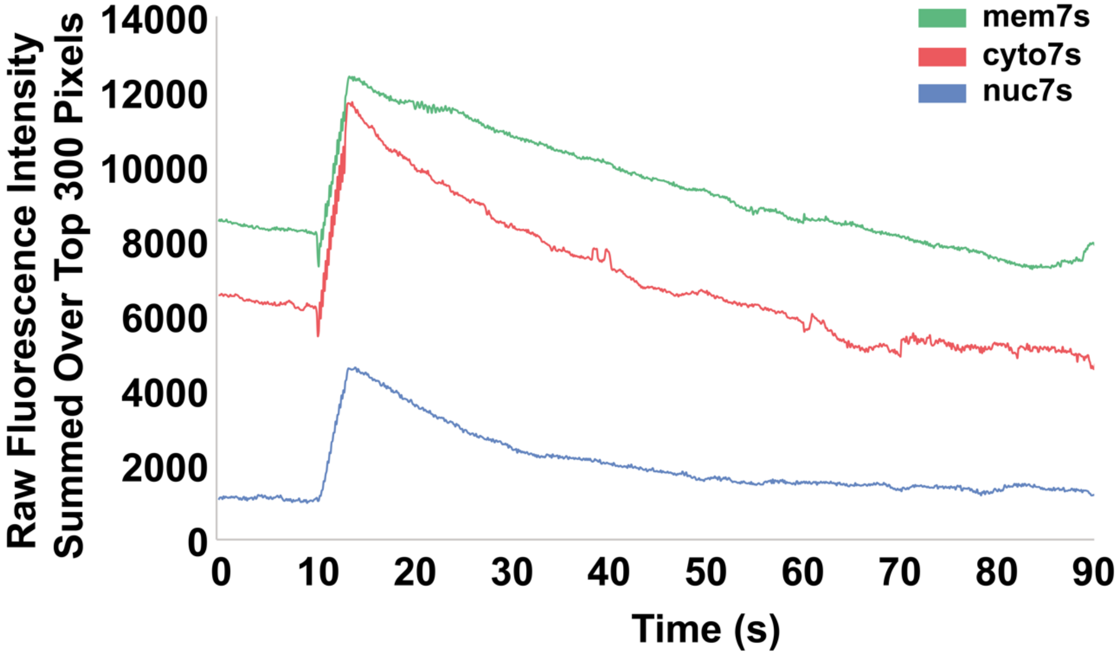

Figure S2: The summed fluorescence intensity of the top 300 pixels for different localizations of GCaMP. Plotted is the raw fluorescence intensity before normalization averaged across all available experimental data.
