# Supplementary material for: An expanded GCaMP reporter toolkit for functional imaging in Caenorhabditis elegans
Source: G3 (Bethesda). 2023 Aug 11;13(10):jkad183. doi: 10.1093/g3journal/jkad183 (PMC10542313; doi:10.1093/g3journal/jkad183)
Supplement: jkad183_Supplementary_Data [file jkad183_supplementary_data.zip › Figure_S3_G3-2023-404350.pdf]

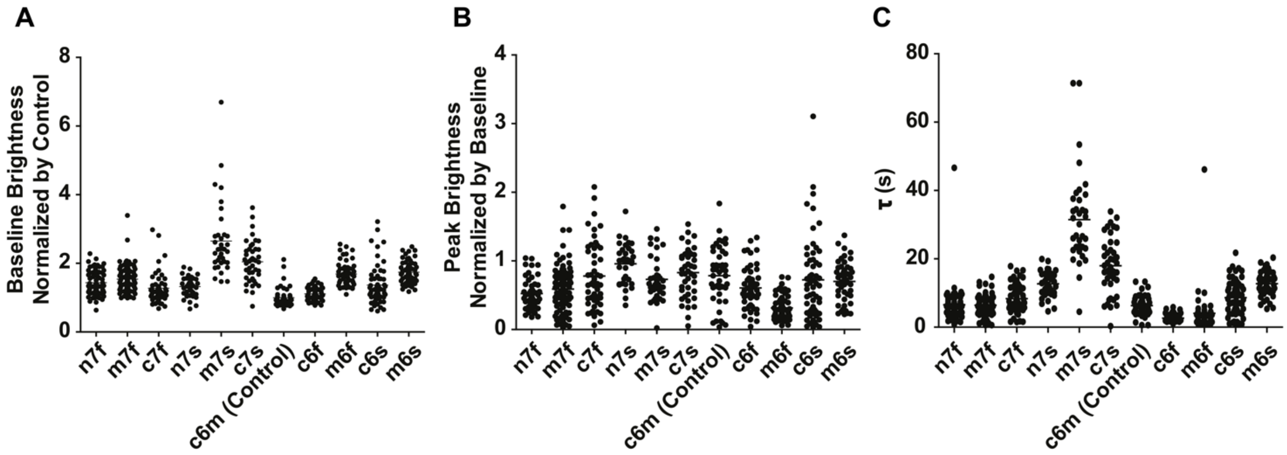

Figure S3: Baseline brightness (A), peak brightness (B), and decay time constants (C) of GCaMP for all GCaMP variants and localizations. The statistical analyses for all distributions are available in the File S1.
