# Supplementary material for: An expanded GCaMP reporter toolkit for functional imaging in Caenorhabditis elegans
Source: G3 (Bethesda). 2023 Aug 11;13(10):jkad183. doi: 10.1093/g3journal/jkad183 (PMC10542313; doi:10.1093/g3journal/jkad183)
Supplement: jkad183_Supplementary_Data [file jkad183_supplementary_data.zip › Figure_S4_G3-2023-404350.pdf]

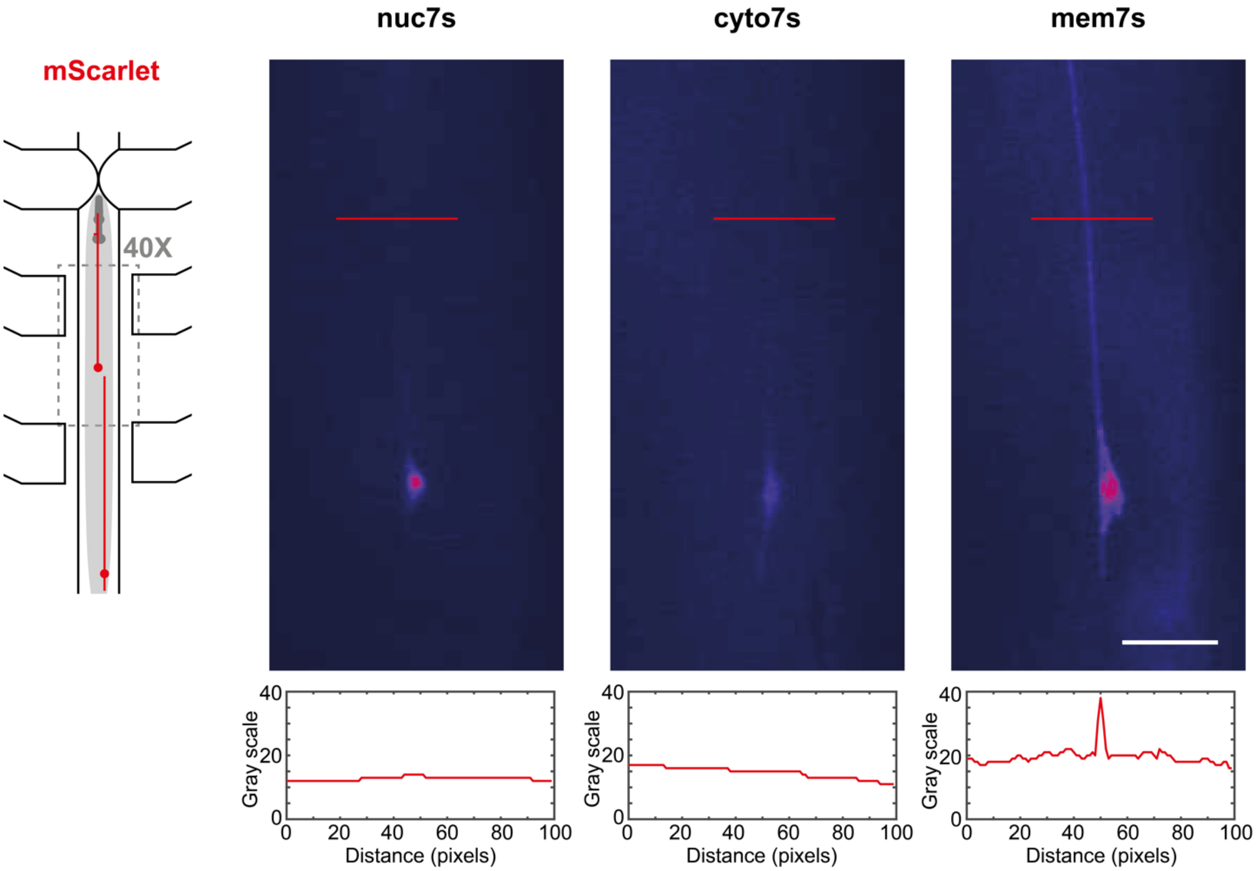

Figure S4: Neurite visibility in different subcellular localized versions of mScarlet expression. The calcium-insensitive mScarlet expression is important for reliable tracking of GCaMP activity in the neurites during both on and off activity states. Neurites are clearly visible in the membrane-localized version, unlike other subcellular localized versions. Scale bar: 25 µm.
