# Supplementary material for: An expanded GCaMP reporter toolkit for functional imaging in Caenorhabditis elegans
Source: G3 (Bethesda). 2023 Aug 11;13(10):jkad183. doi: 10.1093/g3journal/jkad183 (PMC10542313; doi:10.1093/g3journal/jkad183)
Supplement: jkad183_Supplementary_Data [file jkad183_supplementary_data.zip › Table_S1_G3-2023-404350.docx]

**Table S1: Transgenic strains**

| **Strain Name** | **GCaMP variant** | **Subcellular localization** | **Genotype** | **Reference** |
| --- | --- | --- | --- | --- |
| AQ3236 | 6m | Cytosolic | *ljSi2 [mec-7::GCaMP6m::SL2::TagRFP + unc-119(+)] II; unc-119(ed3) III.* | (Cho *et al.* 2017) |
| GT349 | 6s | Membrane | *aSi25[lox2272 Cbr-unc-119(+) lox2272 + mec-7p::GCaMP6s::ras-2CAAX::SL2::mScarlet-I::ras-2CAAX] II; unc-119(ed3) III.* | This work |
| GT350 | 7s | Membrane | *aSi26[lox2272 Cbr-unc-119(+) lox2272 + mec-7p::GCaMP7s::ras-2CAAX::SL2::mScarlet-I::ras-2CAAX] II; unc-119(ed3) III.* | This work |
| GT370 | 6f | Membrane | *aSi29[lox2272 Cbr-unc-119(+) lox2272 + mec-7p::GCaMP6f::ras-2CAAX::SL2::mScarlet-I::ras-2CAAX] II; unc-119(ed3) III.* | This work |
| GT372 | 7f | Membrane | *aSi31[lox2272 Cbr-unc-119(+) lox2272 + mec-7p::GCaMP7f::ras-2CAAX::SL2::mScarlet-I::ras-2CAAX] II; unc-119(ed3) III.* | This work |
| GT324 | 6s | Nuclear Localized | *aSi4[lox2272 Cbr-unc-119(+) lox2272 + mec-7p::NLS::GCaMP6s::egl-13NLS::SL2::NLS::mScarlet-I::egl-13NLS] II; unc-119(ed3) III.* | This work |
| GT330 | 7s | Nuclear Localized | *aSi8[lox2272 Cbr-unc-119(+) lox2272 + mec-7p::NLS::GCaMP7s::egl-13NLS::SL2::NLS::mScarlet-I::egl-13NLS] II; unc-119(ed3) III.* | This work |
| GT346 | 6f | Nuclear Localized | *aSi22[lox2272 Cbr-unc-119(+) lox2272 + mec-7p::NLS::GCaMP6f::egl-13NLS::SL2::NLS::mScarlet-I::egl-13NLS] II; unc-119(ed3) III.* | This work |
| GT347 | 7f | Nuclear Localized | *aSi23[lox2272 Cbr-unc-119(+) lox2272 + mec-7p::NLS::GCaMP7f::egl-13NLS::SL2::NLS::mScarlet-I::egl-13NLS] II; unc-119(ed3) III.* | This work |
| GT374 | 6s | Cytosolic | *aSi33[lox2272 Cbr-unc-119(+) lox2272 + mec7p::GCaMP6s::SL2::mScarlet-I] II; unc-119(ed3) III.* | This work |
| GT375 | 7s | Cytosolic | *aSi27[lox2272 Cbr-unc-119(+) lox2272 + mec-7p::GCaMP7s::SL2::mScarlet-I] II; unc-119(ed3) III.* | This work |
| GT376 | 6f | Cytosolic | *aSi35[lox2272 Cbr-unc-119(+) lox2272 + mec7-p::GCaMP6f::SL2::mScarlet-I] II; unc-119(ed3) III.* | This work |
| GT377 | 7f | Cytosolic | *aSi36[lox2272 Cbr-unc-119(+) lox2272 + mec7-p::GCaMP7f::SL2::mScarlet-I] II; unc-119(ed3) III.* | This work |
