# Supplementary material for: An expanded GCaMP reporter toolkit for functional imaging in Caenorhabditis elegans
Source: G3 (Bethesda). 2023 Aug 11;13(10):jkad183. doi: 10.1093/g3journal/jkad183 (PMC10542313; doi:10.1093/g3journal/jkad183)
Supplement: jkad183_Supplementary_Data [file jkad183_supplementary_data.zip › Table_S2_G3-2023-404350.docx]

**Table S2: Statistical Comparisons for all tested GCaMP Variants**

The Kruskal-Wallis test was performed, followed by Dunn’s multiple comparison test. Each row compares two GCaMP variants and shows the probability that the null hypothesis is true. If the null hypothesis is true, all data of the compared variants are sampled from populations with identical distributions, and therefore, differences between groups are due to random sampling (* P < 0.05, ** P < 0.01, *** P<0.001).

| **Comparison** | **Baseline Brightness** | **Peak Brightness** | **Decay time constant** |
| --- | --- | --- | --- |
| n7s vs n7f | ns | *** | *** |
| n7s vs m7s | *** | ns | ns |
| n7s vs m7f | ns | *** | *** |
| n7s vs c7f | ns | ns | * |
| n7s vs c7s | *** | ns | ns |
| n7s vs m6s (control) | ** | ** | *** |
| n7s vs c6f | ns | ** | * |
| n7s vs c6s | ns | ns | *** |
| n7s vs m6f | ** | *** | *** |
| n7s vs m6s | *** | ns | ns |
| n7f vs m7s | *** | ns | *** |
| n7f vs m7f | ns | ns | ns |
| n7f vs c7f | ns | ns | ns |
| n7f vs c7s | *** | ** | *** |
| n7f vs m6s (control) | *** | ns | *** |
| n7f vs c6f | *** | ns | ns |
| n7f vs c6s | ns | * | ns |
| n7f vs m6f | ns | ns | *** |
| n7f vs m6s | ** | ns | *** |
| m7s vs m7f | *** | ns | *** |
| m7s vs c7f | *** | ns | *** |
| m7s vs c7s | ns | ns | ns |
| m7s vs m6s (control) | *** | ns | *** |
| m7s vs c6f | *** | ns | *** |
| m7s vs c6s | *** | ns | *** |
| m7s vs m6f | ** | *** | *** |
| m7s vs m6s | * | ns | ns |
| m7f vs c7f | ** | ns | ns |
| m7f vs c7s | ** | * | *** |
| m7f vs m6s (control) | *** | ns | *** |
| m7f vs c6f | *** | ns | ns |
| m7f vs c6s | ns | ns | ns |
| m7f vs m6f | ns | *** | *** |
| m7f vs m6s | ns | ns | *** |
| c7f vs c7s | *** | ns | *** |
| c7f vs m6s (control) | ns | ns | *** |
| c7f vs c6f | ns | ns | ns |
| c7f vs c6s | ns | ns | ns |
| c7f vs m6f | *** | *** | *** |
| c7f vs m6s | *** | ns | ** |
| c7s vs m6s (control) | *** | ns | *** |
| c7s vs c6f | *** | ns | *** |
| c7s vs c6s | *** | ns | *** |
| c7s vs m6f | ns | *** | *** |
| c7s vs m6s | ns | ns | ns |
| m6s (control) vs c6f | ns | ns | *** |
| m6s (control) vs c6s | ** | ns | *** |
| m6s (control) vs m6f | *** | *** | ns |
| m6s (control) vs m6s | *** | ns | *** |
| c6f vs c6s | ns | ns | ns |
| c6f vs m6f | *** | *** | *** |
| c6f vs m6s | *** | ns | ** |
| c6s vs m6f | *** | *** | ** |
| c6s vs m6s | *** | ns | *** |
| m6f vs m6s | ns | *** | *** |

.
